# Supplementary material for: β-blockers augment L-type Ca2+ channel activity by targeting spatially restricted β2AR signaling in neurons
Source: eLife. 2019 Oct 14;8:e49464. doi: 10.7554/eLife.49464 (PMC6813027; doi:10.7554/eLife.49464)
Supplement: Supplementary file 1. — Values are mean ± SEM. *p<0.05 with Kruskal Wallis – Dunn’s multiple comparison test. [file elife-49464-supp1.docx]

|  | NT | ISO | CAR | CAR_out | TIM |
| --- | --- | --- | --- | --- | --- |
| P_o_ (%) | 8.2 ± 1.9 | 40.8 ± 4.0* | 30.2 ± 4.8* | 15.2 ± 3.5 | 11.0 ± 4.1 |
| nP_o_ (%) | 8.2 ± 1.9 | 52.1 ± 7.1* | 34.7 ± 5.7* | 15.2 ± 3.5 | 11.3 ± 4.0 |
| n (# channels) | 2.6 ± 0.5 | 4.0 ± 0.6 | 2.8 ± 0.2 | 1.9 ± 0.1 | 2.4 ± 0.3 |
| availability (%) | 39.2 ± 9.7 | 75.6 ± 7.5* | 64.7 ± 4.1 | 41.1 ± 5.5 | 38.4 ± 7.6 |
| I _mean ensemble avg_ (fA) | 142.0 ± 38.6 | 308.0 ± 37.2* | 304.6 ± 27.2* | 189.3 ± 33.7 | 154.9 ± 28.8 |
| sweeps | 867 | 1001 | 2058 | 1187 | 998 |
| N (# patches) | 9 | 11 | 22 | 12 | 10 |

**Supplementary file 1. Biophysical properties of L-type Ca^2+^ currents in neurons recorded in Figure 5A-5E.** Values are mean ± SEM. **P* < 0.05 with Kruskal Wallis – Dunn’s multiple comparison test.
