# Supplementary material for: β-blockers augment L-type Ca2+ channel activity by targeting spatially restricted β2AR signaling in neurons
Source: eLife. 2019 Oct 14;8:e49464. doi: 10.7554/eLife.49464 (PMC6813027; doi:10.7554/eLife.49464)
Supplement: Supplementary file 2. — Values are mean ± SEM. *p<0.05 with Kruskal Wallis – Dunn’s multiple comparison test. [file elife-49464-supp2.docx]

**Supplementary file 2. Biophysical properties of L-type Ca^2+^ currents in the neurons recorded in Figure 7C-7G.** Values are mean ± SEM. **P* < 0.05 with Kruskal Wallis – Dunn’s multiple comparison test.

|  | mutant | mutant + ISO | mutant + CAR |
| --- | --- | --- | --- |
| P_o_ (%) | 10.0 ± 2.8 | 10.3 ± 3.3 | 26.5 ± 4.3* |
| nP_o_ (%) | 10.7 ± 2.9 | 11.5 ± 3.9 | 35.3 ± 7.1* |
| n (# channels) | 2.7 ± 0.5 | 2.5 ± 0.2 | 2.9 ± 0.3 |
| availability (%) | 37.8 ± 8.1 | 36.3 ± 4.5 | 63.2 ± 4.5* |
| I _mean ensemble avg_ (fA) | 174.1 ± 38.9 | 157.5 ± 34.4 | 417.0 ± 41.4* |
| sweeps | 1128 | 1164 | 1740 |
| N (# patches) | 12 | 11 | 18 |
